# Change in sex hormone profile due to exposure to nuclides in nuclear detonation crisis: a topic to be discussed in reproductive medicine

**Published:** 2012-05

**Authors:** Viroj Wiwanitkit

**Affiliations:** *Wiwanitkit House, Bangkhae, Bangkok Thailand.*

Dear Editor,

The big public health concern in this year is the nuclear crisis in Japan that causes contamination of nuclides around the world. The effect of the exposure to leaked nuclides from the present nuclear crisis on reproductive health is a topic to be concerned. In my previous publication (1), the evidence on the relationship between nuclear exposure and infertility can be confirmed. However, there are also other adverse effects on the reproductive system of the exposed subjects. 

An interesting topic is the disturbance of the normal sex hormone system. There are some reports on the effect of exposure on sex hormone profile in exposed subjects. Based on the data from the referencing nuclear crisis, the Chernobly crisis, the change in the level of testosterone in exposed subjects were controversial. Some reports mentioned for low testosterone level (2) while the others noted for rising level (3-5) among exposed subjects.

However, more reports points to the increasing level of testosterone. Nevertheless, the observation on the increased testosterone level in adolescent offspring of exposed subjects was also reported (5). Therefore, the problem of testosterone hormone alteration is a problem in reproductive health of the exposed subjects and this might lead to infertility (1). Hence, this is an interesting topic to be studied in the present Fukushima crisis. Unlike the report on testosterone, there are few reports on estrogen. The interesting report is on the estrogen related cancers in females exposed to the nuclides in the Chernobyl crisis. The study on breast cancers revealed that “BRCA1 mutations were strongly associated with earlier age at diagnosis, with estrogen receptor (ER) negative tumors (6).”

However, there is a report on the change of estrogen and progesterone in animals in the contaminated areas (7). For FSH and LH, although FSH and LH are not the actual sex hormones from gonad, the change of FSH and LH is another interesting issue. According to the study of Goncharov *et al* (4), there was a significant lowering of LH level but there was no significant change in FSH. However, in other studies, the increased FSH indicating the infertility was observed (2, 8). 

In conclusion, there are evidences on sex hormone change in the subjects exposed to nuclides from nuclear accident crisis. This is an important issue in reproductive medicine that should be the focus in following up of the exposed subjects in the present nuclear crisis.
